# EHMTI-0098. Frontal executive functions in medication overuse headache

**DOI:** 10.1186/1129-2377-15-S1-C48

**Published:** 2014-09-18

**Authors:** HA Sahin, Y Turkel, EO Sahin, T Ozbenli

**Affiliations:** 1 Neurology, Ondokuz Mayis University Faculty of Medicine, Samsun, Turkey; 2 Neurology, Education ve Research Hospital, Samsun, Turkey; 3 Neurology, Ondokuz Mayis University Faculty of Medicine, Samsun, Turkey

## Introduction

In the neuroimaging studies, prefrontal dysfunctions have been reported in the patients with medication overuse headache (MOH).

## Aims

The study investigates the presence of cognitive deficits in patients with medication overuse headache (MOH) or migraine without aura as compared to healthy controls.

## Methods

Neuropsychological test battery assessing frontal executive functions and attention was applied in 50 patients with MOH, 50 patients with migraine without aura and 50 control subjects. Depressive symptoms were measured with Hamilton depression rating scale.

## Results

The results were compared between the three groups using analysis of covariance. Tukey test was used for post hoc multiple comparisons. The patients with MOH performed worse than patients with migraine on the digit span-forward, digit span-backward, verbal fluency KAS, animal numbers in attention and total categories completed on WCST, TMT A-B, total number of errors on CPT in executive functions whereas they were worsen than the control subjects on almost all tests. The patients with migraine were significantly poorer than control subjects on digit span-backward, and showed higher number of perseverating responses on WCST, total number of errors on CPT, and total number of errors on Go-NoGo.

## Conclusions

The patients with MOH have frontal dysexecutive syndrome characterized by an inability in the inhibition of inappropriate responses and attention deficit. There are studies suggesting an association between drug addiction behavior and MOH via prefrontal cortex involvement. Our results further support the notion that MOH may be a part of the spectrum of drug addiction behavior.

No conflict of interest.

